# Macrophages‐derived small extracellular vesicles regulate chondrocyte proliferation and affect osteoarthritis progression via upregulating Osteopontin expression

**DOI:** 10.1002/ccs3.70008

**Published:** 2025-04-22

**Authors:** Min Tu, An‐Min Liu, Wei Huang, Dan Wang, Hou‐Qiong Chen, Xiao‐Yuan Hu

**Affiliations:** ^1^ Department of Orthopedics The People's Hospital of Jingmen Jingmen Hubei China; ^2^ Jingmen People's Hospital Affiliated to Jingchu University of Technology Jingmen Hubei China

**Keywords:** M2 macrophages, osteoarthritis, osteopontin (OPN), small extracellular vesicles (sEVs)

## Abstract

Small extracellular vesicles (sEVs) are considered promising gene‐delivery vehicles for the treatment of osteoarthritis (OA). This study aimed to explore the molecular mechanism by which M2 macrophage‐derived sEVs (M2‐sEVs) modulate chondrocyte proliferation and apoptosis, thereby affecting OA progression. M2 macrophages were successfully induced, and M2‐sEVs were successfully isolated. The sEVs were small vesicles with diameters ranging from 50 to 150 nm. The exosomal markers, including CD9, CD63, and CD81, were highly expressed, whereas the negative marker calnexin was absent in M2‐sEVs. M2‐sEVs effectively alleviated OA tissue and chondrocyte damage in both in vivo and in vitro models, evidenced by reduced rat knee joint injury, increased chondrocyte viability, and decreased chondrocyte apoptosis and extracellular matrix (ECM) degradation. Furthermore, M2‐sEVs decreased the levels of pro‐inflammatory cytokines IL‐6 and TNF‐α. Osteopontin (OPN) was upregulated within rats with OA and IL‐1β‐induced chondrocytes. Silencing of OPN exacerbated IL‐1β‐induced chondrocyte damage and partially abrogated the therapeutic effects of M2‐sEVs. Additionally, M2‐sEVs enhanced OPN expression and activated CD44 and the PI3K/AKT signaling pathway. In conclusion, M2‐sEVs promoted OPN expression to improve knee joint tissue damage in rats with OA and chondrocyte damage. This protective effect of M2‐sEVs might be associated with the activation of CD44 and the PI3K/AKT signaling.

## Graphical abstract

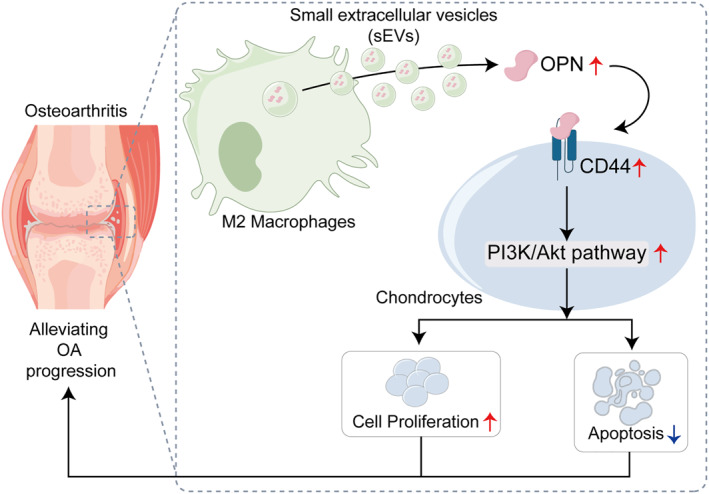

## 1 INTRODUCTION

Osteoarthritis (OA) is one of the most prevalent orthopedic disorders.^1^ It is primarily marked by articular cartilage injury which is not confined to cartilage but extends to the entire joint structure, ultimately leading to degenerative changes, fibrosis, and articular cartilage fractures.^2^ Statistical data indicate that OA affects approximately 8%–12% of the global population aged 50 and above, with prevalence rising to over 50% among individuals aged 65 and older.^3^ Despite its widespread impact, the molecular mechanisms underlying OA pathogenesis remain poorly understood. As previously reported, gene regulation in chondrocytes and inflammatory responses in the progression of OA play crucial roles in OA development.^4^

Small extracellular vesicles (sEVs) are extracellular vesicles containing DNA, RNA, and proteins, and they play a critical role in intercellular communication. These vesicles are emerging as pivotal players in a wide range of physiological and pathological processes including immune response, inflammation, tumor growth, and infection.^5^, ^6^, ^7^ The role of sEVs in OA has also gained attention. For example, bone marrow‐derived extracellular vesicles is shown to alleviate knee joint pain in OA rat models.^8^ Macrophage polarization exerts a crucial effect on inflammatory diseases including OA. Research has demonstrated a strong correlation between macrophage polarization and inflammation during OA progression.^9^ In recent years, growing studies have provided evidence for the effect of macrophage‐secreted sEVs on various inflammatory disorders. For example, sEVs secreted by bone marrow‐derived macrophages can regulate inflammatory responses and ameliorate atherosclerosis.^10^ Similarly, M2 macrophage‐derived extracellular vesicles encapsulated in hyaluronic acid were reported to alleviate OA by modulating macrophage polarization.^11^ Conversely, macrophage‐derived extracellular vesicles were implicated in triggering noncanonical pyroptosis in chondrocytes, thereby promoting cartilage catabolism in OA.^12^ Nevertheless, the specific effect and mechanism of M2 macrophage‐derived sEVs (M2‐sEVs) in OA pathogenesis remain unclear.

Osteopontin (OPN), commonly referred to as early T cell‐activation gene 1, is a multifunctional phosphoprotein.^13^ OPN can be produced by multiple cellular types, including macrophages, lymphocytes, and epithelial cells.^14^ It was indicated that OPN is tightly implicated in OA pathogenesis.^15^ Although OPN expression is elevated within the synovial fluid and cartilage tissues of OA patients,^16^ studies suggest that OPN might alleviate OA progression by promoting chondrocyte anabolism,^15^ facilitating chondrocyte proliferation,^17^ and inhibiting chondrocyte apoptosis.^18^ Additionally, M2‐sEVs were revealed to induce bone differentiation and upregulate OPN expression.^19^ Based on these findings, it is hypothesized that M2 macrophages may mediate the modulation of OPN expression to contribute to the alleviation of OA.

Herein, M2‐sEVs were prepared and evaluated for their therapeutic effects on OA in both in vitro and in vivo models. In summary, M2‐sEVs can alleviate cartilage injury and inflammation in cartilage tissues of rat knee joint, inhibit chondrocyte apoptosis, and promote OPN expression. These results might provide a novel research direction for treating OA with M2‐sEVs.

## 2 MATERIALS AND METHODS

### 2.1 Ethical statement

All experimental procedures and animal treatments were carried out under the approval of the Jingmen People's Hospital Medical Ethics Committee (approval No.: KY‐202304050002) and conducted following the Guide for the Care and Use of Laboratory Animals of the National Institutes of Health.

### 2.2 Isolation and characterization of M2 macrophages from rats

The femur and tibia of SD rats were collected with both ends cut to open the bone cavity. The bone marrow was washed through injecting sterile phosphate‐buffered saline (PBS) with a 10‐ml syringe. Next, red blood cell lysate was added for resuspension followed by lysis on ice for 10 min. After resuspension in a complete culture medium, recombinant rat macrophage colony‐stimulating factor (M‐CSF; 10 ng/ml, PeproTech, Rocky Hill, USA) was used to induce monocytes to differentiate into macrophages. After culture for 7 days, a complete culture medium containing 10 ng/ml interleukin (IL‐)4 (PeproTech) was used to replace the media for 48 h to induce differentiation into M2 macrophages.

For M2 macrophage identification, FITC‐labeled CD206 antibody (sc‐58986; Santa Cruz Biotech, Santa Cruz, USA) was analyzed using flow cytometry, and Arginase and CD206 mRNA levels were detected using qRT‐PCR.

### 2.3 Isolation and confirmation of M2‐sEVs

After 72‐h culturing (37°C, 5% CO_2_) of M2 macrophages in a serum‐free culture medium (for exosome, Yesen) within a humidified atmosphere, the culture media was centrifuged (2000 g, 20 min) followed by further centrifugation (10,000 g, 30 min) at 4°C. Additionally, the liquid portion was filtered through a pore size of 0.22 μm. Isolation of M2‐sEVs was conducted through ultracentrifugation (100,000 g, 120 min, 4°C).^20^ The exosome pellet was eventually resuspended and stored for subsequent analyses. TEM was employed to confirm the isolated exosomes. The exosome content and size distribution were measured using NTA with the NanoSight NS300 instrument. The level of exosomal markers [CD9 (1:1000, 20597‐1‐AP, Proteintech, Wuhan, China), CD63 (1:5000, 67605‐1‐Ig, Proteintech), and CD81 (1:1000, 27855‐1‐AP, Proteintech)] as well as negative control calnexin (1:5000, 10427‐2‐AP, Proteintech)^21^ was determined using Western blot.

Based on protein contents determined using a Pierce™ bicinchoninic acid (BCA) protein assay kit (Thermo Fisher Scientific, Waltham, USA), rat chondrocytes (2 × 10^5^ cells) were subjected to 24‐h treatment with M2‐sEVs at 50 μg/ml.^22^

### 2.4 Establishment of animal model

Eight‐week‐old healthy male SPF SD rats were procured from the SLAC laboratory animal company (Hunan, China). All rats were adaptively fed for 1 week before the formal experiment. OA was induced within the rats' right knee using destabilized medial meniscus (DMM) surgery. In short, after inhalation anesthesia with isoflurane, rat skin was prepared and the surgical area skin was disinfected with iodine. The rat was placed in a supine position, and the joint capsule medial to the patellar tendon of the right knee was incised. After surgically sectioning the medial meniscus‐tibial plateau ligament with micro‐surgical scissors under a microscope, the meniscus was removed. Next, the joint capsule, medial thigh muscle, and connective tissues were closed sequentially with absorbable sutures, and the surgical area skin was then closed with nonabsorbable sutures. The right knee was disinfected with iodine. Rats were postoperatively administrated with ampicillin to prevent infection. The control rats were only subjected to opening the right knee joint capsule without sectioning the medial meniscus‐tibial ligament. Rats were designated at random into five groups (*n* = 6): normal, sham, sham + PBS, OA, and OA + M2‐sEVs groups. Specifically, the normal rats were fed normally and not treated in any way; the sham group rats underwent surgery but without DMM; the sham + PBS rats and rats with OA were subjected to injection with an equal amount of PBS into the joint cavity; starting from the fourth week of the experiment, a micro‐syringe was employed to inject intra‐articularly the rats with OA + M2‐sEVs with 10 μL of M2‐sEVs (10^10^ particles/mL) every five days for four consecutive injections.^23^ Subsequently, rats were fed in a standard environment (12 h light/12 h dark cycles, 25°C, 60% humidity) and accessed to food and water ad libitum. Four weeks after the first injection of M2‐sEVs, rats were euthanized with their right knee joint harvested for the subsequent experiments.

### 2.5 Hematoxylin and eosin (H&E) staining and saffron O/fast green staining

The cartilage tissues of rat knee joints were fixed with 4% paraformaldehyde and decalcified using 10% ethylene diamine tetra‐acetic acid (EDTA). The paraffin‐embedded tissue samples were cut into 5‐μm slices. Next, slices were deparaffinized using xylene (Sinopharm Chemical Reagent Co., Ltd., Shanghai, China) and hydrated in gradient ethanol. For H&E staining, slices were subjected to 10‐min staining with hematoxylin (Servicebio, Wuhan, China), rinsed in tap water, and then immersed (5 s) in hydrochloric acid ethanol. After tap water washing, the sections were stained (10 min) with eosin (Servicebio) and then washed in tap water. Next, slices were subjected to immersion within 70%, 80%, and 90% alcohol for 10 s each, and then in anhydrous ethanol for 10 s. Finally, the sections were cleared (10 min) in xylene before being sealed with neutral resin.

For safranin‐O/fast green staining, slices were subjected to 5‐min staining with fast green stain solution (Servicebio) and rinsed with tap water to remove excess dye solution. After treatment with 1% hydrochloric acid for 15 s and tap water washing, the sections were stained (5 s) with safranin (Servicebio), and then quickly dehydrated with anhydrous ethanol. The dehydration process was repeated 4 times, 3–5 s for each time. Finally, following 10‐min xylene clearance, the neutral resin was used to seal slices, and a microscope (Olympus, Tokyo, Japan) was employed to observe slices. The Osteoarthritis Research Society International (OARSI) score^24^ was utilized to evaluate the cartilage degeneration degree of the rat knee joint.

### 2.6 Terminal deoxynucleotidyl transferase (TdT)‐mediated dUTP Nick end labeling (TUNEL) staining

A TUNEL kit (Servicebio) was employed to detect the apoptosis of the cartilage tissues of the rat knee joint. After dewaxing and hydration, slices were subjected to treatment with 20 μg/ml protease K for antigen repair (37°C, 20 min). Next, the sections were incubated (room temperature, 20 min) with 3% hydrogen peroxide in a dark environment, and then added with 50 μL TUNEL working solution (KeyGene, Nanjing, China) for further incubation (37°C, 30 min). Slices were rinsed in PBST, and then incubated with 2,4‐diaminobutyric acid (DAB) in converter‐peroxide for 30 min, followed by re‐staining with hematoxylin. An optical microscope (Olympus) was used for observation. The nucleus of apoptotic cells was brown.

### 2.7 Cell culture and treatment

Rat chondrocytes were purchased from Procell (CP‐R087; Wuhan, China) and were cultivated within DMEM (Thermo Fisher Scientific) supplemented with 10% fetal bovine serum (FBS) and 100 U/mL penicillin/streptomycin. An OA cell model was induced by 24‐h stimulation of rat chondrocytes with 10 ng/ml IL‐1β. After 24‐h IL‐1β stimulation, cells were subjected to further 24‐h treatment with 50 μg/mL M2‐sEVs or M2‐GW‐sEVs [sEVs were isolated from the conditioned medium obtained after culturing M2 macrophages with 20 μg/ml GW4869 (blocking the generation of sEVs)].^22^

### 2.8 Cell transfection

GenePharma (Shanghai, China) was applied to construct small interfering RNA (siRNA) mediated OPN (si‐OPN) and its negative control (si‐NC). The sequences of si‐OPN are listed in Table 1. Rat chondrocytes were subjected to transfection with si‐OPN/si‐NC using OriTrans®PEI‐X (ORI2311, ORI‐BIO, Changsha, China). Cell transfection was performed after 24‐h IL‐1β induction in chondrocytes.

**TABLE 1:** qRT‐PCR primer sequence and siRNA sequences (Rats).

Primers		Sequences (5′‐3′)
Arginase	Forward	AGACAGGGCTACTTTCAGGACTA
	Reverse	TTATGATTACCTTCCCGTTTCG
CD206	Forward	ATGAGACTCCCCCTGCTCCTGG
	Reverse	CTGAACGGAGATGGCGCTTAGAG
OPN	Forward	GAGGTGATAGCTTGGCTTACGG
	Reverse	CAGACGCTGGGCAACTGGGATG
GAPDH	Forward	GCCTTCCGTGTTCCTACCCC
	Reverse	CGCCTGCTTCACCACCTTCT
si‐OPN	Forward	AGCUAGUCCUAGACCCUAATT
	Reverse	UUAGGGUCUAGGACUAGCUTT
si‐NC	Forward	UUCUCCGAACGUGUCACGUTT
	Reverse	ACGUGACACGUUCGGAGAATT

### 2.9 Quantitative real‐time polymerase chain reaction (qRT‐PCR)

The TRIzol reagent (Thermo Fisher Scientific) was employed to isolate total RNA from rat right knee joint tissues, rat chondrocytes, and macrophages. A multifunctional microplate reader (HeaLForce, Hong Kong, China) was utilized to determine RNA content. A reverse transcription kit (GenStar, Beijing, China) was used to reverse transcribe total RNA into cDNA. BeyoFast™ SYBR Green qPCR Mix (Bio‐Rad, Hercules, USA) was employed to perform qPCR upon the qTower 3G fluorescence quantitative PCR instrument (Analytic Jena, Jena, Germany). The primer sequences used in qRT‐PCR are shown in Table 1. GAPDH was used for the normalization of the relative expression of target genes which was calculated using the 2^−ΔΔCt^ method.

### 2.10 Western blot

RIPA lysis buffer (Beyotime, Shanghai, China) was employed to isolate total proteins from rat knee joint cartilage tissue samples and chondrocytes, and the BCA protein concentration detection kit (Beyotime) was used to determine the protein content. Next, following electrophoresis by sodium dodecyl sulfate‐polyacrylamide gel electrophoresis (SDS‐PAGE), the separated proteins were electroblotted from the gel onto polyvinylidene fluoride (PVDF) membranes (Merck Millipore, Billerica, USA). Membranes were blocked using 5% nonfat milk for 1 h followed by an overnight incubation at 4°C using the primary antibodies anti‐collagen II (1:2000, ab34712, Abcam, Cambridge, USA), anti‐matrix metalloproteinase (MMP)‐3 (1:2000, 17873‐1‐AP, Proteintech), anti‐MMP‐13 (1:2000, AF5355, Affinity Bioscience, Changzhou, China), anti‐bax (1:5000, 50599‐2‐Ig, Proteintech), anti‐Bcl‐2 (1:2000, 68103‐1‐Ig, Proteintech), anti‐cleaved caspase 3 (1:2000, AF7022, Affinity Biosciences), anti‐total caspase 3 (1:2000, 19677‐1‐AP, Proteintech), anti‐OPN (1:2000, 22952‐1‐AP, Proteintech), anti‐CD44 (1:2000, 15675‐AP, Proteintech), anti‐p‐PI3K (1:2000, AF3242, Affinity Bioscience), anti‐PI3K (1:2000, 60225‐1‐Ig, Proteintech), p‐AKT (1:2000, 66444‐1‐Ig, Proteintech), anti‐AKT (1:2000, 10176‐2‐AP, Proteintech), and anti‐GAPDH (1:2000, AF7021, Affinity Bioscience). Membranes were washed in PBS, followed by 2‐h incubation at room temperature (RT) using horseradish peroxidase‐labeled goat anti‐rabbit IgG antibodies (1:5000, GAR007, Multi‐Sciences, Hangzhou, China) and goat anti‐mouse IgG antibodies (1:5000; GAM007; Multi‐Sciences). An enhanced chemiluminescence (ECL) kit (Beyotime) was applied to visualize proteins. GAPDH was utilized as an internal reference. ImageJ (National Institutes of Health, Bethesda, USA) was applied to quantify proteins.

### 2.11 Enzyme‐linked immunosorbent assay (ELISA)

ELISA kits were used to detect IL‐6 and tumor necrosis factor‐α (TNF‐α) levels in rat knee joint cartilage tissues and chondrocytes according to the protocols of the manufacturer. ELISA kits for IL‐6 (E‐EL‐R0015) and TNF‐α (E‐EL‐R2856) were procured from Elabscience (Wuhan, China). A BCA kit was employed to determine protein content. Cytokine levels were normalized to per mg protein.

### 2.12 Verification of the uptake of M2‐sEVs by chondrocytes

M2‐sEVs were coated with a PKH26 dye (Sigma‐Aldrich, St. Louis, USA) for labeling as previously mentioned.^25^ In short, First, 30 μL of M2‐sEVs was diluted in 1 mL of diluent C and 6 μL of PKH26 dye for 5 min. Then, 10% bovine serum albumin (BSA) was added to neutralize the excess dye, followed by washing in PBS for 60 min. Finally, chondrocytes were treated with the labeled exosomes. After cultivation (37°C, 5% CO_2_) within a humidified atmosphere for 0.5 , 4, and 24 h, respectively, the chondrocytes were washed thrice in PBS, and fixed (ambient temperature, 10 min) using 4% paraformaldehyde solution. The nuclei were stained using 4′,6‐diamidino‐2‐phenylindole (DAPI; Life Technologies, Gaithersburg, USA). Lastly, following three washings in PBS, a fluorescence microscope (Olympus) was employed to observe cells.

### 2.13 Cell counting kit‐8 (CCK‐8) assay

The CCK‐8 kit was used to detect cell viability according to the protocols of the manufacturer. The treated chondrocytes were planted (5 × 10^3^ cells/well) into 96‐well plates for culture for 0, 24, 48, and 72 h, respectively. Next, 10 μL of CCK‐8 solution (Sigma‐Aldrich) was added into each well followed by a 2‐h incubation at 37°C. Lastly, a multifunctional microplate reader (PerkinElmer, Waltham, China) was applied to determine the optical density (OD) value at a wavelength of 450 nm.

### 2.14 Colony formation assay

The chondrocytes were seeded (10^3^ cells/well) onto six‐well plates and then incubated overnight (37°C, 5% CO_2_). Following 24‐h IL‐1β induction, chondrocytes were subjected to 24‐h treatment with 50 μg/mL M2‐sEVs/M2‐GW‐sEVs. The culture media was refreshed every 2–3 days. Two weeks later, after 20‐min fixation with 4% paraformaldehyde, chondrocytes were subjected to 30‐min staining with 0.1% crystal violet (Servicebio). A digital camera was applied to capture images. Cell colonies in each well were counted and analyzed using ImageJ software.

### 2.15 Flow cytometry

The Annexin V‐FITC/propidium iodide (PI) double staining kit (Keygen) was used to detect chondrocyte apoptosis following the instructions of the manufacturer. In short, The IL‐1β‐induced chondrocytes were subjected to treatment with 50 μg/mL M2‐sEVs/M2‐GW‐sEVs followed by 24‐h incubation. Chondrocytes were then collected, detached with trypsin (NCM Biotech, Suzhou, China), and prepared into cell resuspension (1 × 10^5^ cells) using PBS. After centrifugation at 500g for 5 min, with the supernatant discarded, cells were added and re‐suspended with 500 μL binding buffer. Next, 5 μL Annexin V‐FITC and PI (Keygen) were added, followed by a 15‐min reaction at RT away from light. A flow cytometer (ACEA Pharma, Hangzhou, China) was applied to detect cell apoptosis within 1 h.

### 2.16 Statistical analysis

All obtained experimental data were presented in terms of mean ± SD. Using the GraphPad Prism 9.0 software, the *t*‐test and one‐way analysis of variance (ANOVA) followed by the Tukey method were employed for comparisons among groups. A *p*‐value of less than 0.05 indicates a statistically significant difference.

## 3 RESULTS

### 3.1 Isolation and identification of M2 macrophage‐derived small extracellular vesicles (M2‐sEVs)

**FIGURE 1:**
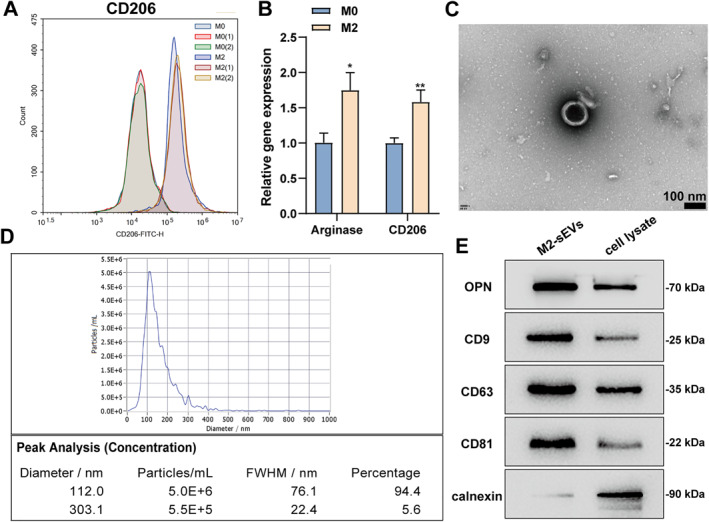
M2‐sEVs was isolated and identified. Rat monocytes were subjected to the 7‐day treatment with M‐CSF to elicit differentiation into M0 macrophages followed by induction for differentiation into M2 macrophages by continuous 48‐h treatment with 10 ng/ml IL‐4. (A) CD206 expression level in M0 and M2 macrophages was detected using flow cytometry. (B) Arginase and CD206 mRNA expressions in M0 and M2 macrophages were detected using qRT‐PCR; exosomes were extracted from M2 macrophages. (C) TEM was applied to observe M2‐sEVs morphology and size; scale bar = 100 nm. (D) the particle size distribution of M2‐sEVs was examined using DLS (E) Western blot was employed to detect OPN, CD9, CD63, CD81, and calnexin expressions in M2‐sEVs and cell lysate of M2 macrophages. *p < 0.05 and **p < 0.01, versus the M0 group.

Monocytes were induced to differentiate into macrophages using M‐CSF, and macrophages were induced to differentiate into M2 type using IL‐4. Flow cytometry was employed to detect the M2 macrophage marker CD206 for identifying M2‐type macrophages. As shown by the results, after induction, the proportion of CD206‐positive cells exceeded 90% (Figure 1A). Moreover, Arginase and CD206 expression levels within M0 and M2 macrophages were detected using qRT‐PCR. The results revealed that after induction for differentiation, M2 macrophages exhibited remarkably higher expressions of Arginase and CD206 than M0 macrophages (Figure 1B). Subsequently, M2‐sEVs were isolated and collected. As shown by TEM observation results, the isolated exosomes were round with 50–150 nm in diameter (Figure 1C). DLS analysis results demonstrated that the size distribution diameter of exosomes was primarily 112.0 nm (Figure 1D). The exosomal markers were further confirmed. In short, protein samples were extracted from M2‐sEVs and the cell lysate of M2 macrophages, respectively. Western blot results demonstrated that the exosomal markers, including CD9, CD63, and CD81, and OPN were remarkably upregulated within the M2‐sEVs, and the negative marker calnexin showed no expression (Figure 1E). The above results indicated the induction of M2 macrophages and the successful isolation of M2‐sEVs.

### 3.2 M2‐sEVs improved OA progression in rats

**FIGURE 2:**
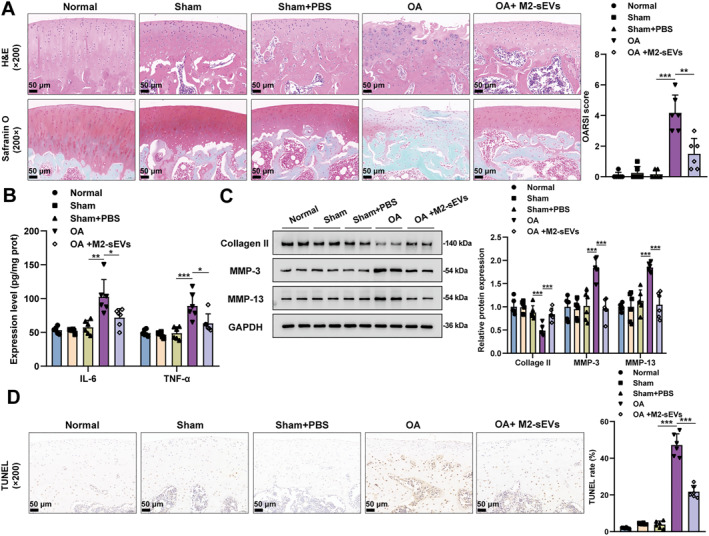
M2‐sEVs improved OA progression in rats. OA was induced within the rats' right knee using destabilized medial meniscus (DMM) surgery. Rats were designated at random into five groups (*n* = 6): normal, sham, sham + PBS, OA, and OA + M2‐sEVs groups. (A) The injury of rat right knee joint tissues was evaluated using H&E and safranin‐O/fast green staining as well as OARSI score; scale bar = 50 μm. (B) IL‐6 and TNF‐α expression levels within the knee joint tissues of rats were detected using ELISA. (C) Collagen II, MMP‐3, and MMP‐13 protein contents within the knee joint tissue samples of rats were detected using Western blot. (D) The rats' knee joint tissue apoptosis was detected using TUNEL staining; scale bar = 50 μm. *p < 0.05, **p < 0.01, and ***p < 0.001.

A rat with OA model was established by using DMM surgery. M2‐sEVs were administrated into rats with OA to assess their therapeutic effects. The rat cartilage tissue morphology and structure were evaluated using H&E and safranin‐O/fast green staining. The results showed that the normal and sham group rats exhibited smooth and complete cartilage tissue surface, neatly arranged chondrocytes, and evenly stained (red) chondrocytes; bone tissues were green with a clear layer. However, in the group of rats with OA, the cartilage tissue surface showed to be rough and chondrocyte arrangement was disordered with light‐stained cartilage matrix and blurred boundaries between the cartilage matrix and bone tissues. These pathological damages of the cartilage tissues of rats with OA can be effectively improved by M2‐sEVs treatment. Additionally, the rats with OA also showed a dramatically higher OARSI score of knee joint specimens than the normal or sham group rats, and M2‐sEVs treatment could significantly reduce the OARSI score of rats with OA (Figure 2A). It is well‐established that pro‐inflammatory factors are important medium of inflammatory response, and aberrant secretion of inflammatory factors will disrupt cartilage matrix metabolism and increase cell apoptosis, eventually leading to cartilage degeneration.^26^ Therefore, ELISA was employed to detect the expressions of proinflammatory mediators (IL‐6 and TNF‐α) within rat knee tissue samples, thus analyzing the anti‐inflammatory effects of M2‐sEVs upon rats with OA. Reportedly, IL‐6 and TNF‐α expressions were notably increased within the knee tissues of rats with OA relative to the normal or sham group; M2‐sEVs treatment could effectively attenuate the elevation of these mediators (IL‐6 and TNF‐α) within the rats with OA (Figure 2B). On the other hand, it was shown that the imbalance between the synthesis and degradation within cartilage matrix greatly participates in cartilage degradation within OA in which MMPs play a decisive role.^27^ Western blot was carried out to detect the extracellular matrix (ECM)‐related protein levels in rat knee tissues. Taken together, as compared to normal or sham group, rats with OA showed notably decreased Collagen II levels and increased the levels of MMP‐3 and MMP‐13 within the knee tissues; M2‐sEVs treatment could improve these changes in rats with OA as manifested by the remarkably elevated Collagen II level and reduced MMP‐3 and MMP‐13 levels (Figure 2C). The knee joint tissue apoptosis was detected using TUNEL staining, suggesting that rats with OA exhibited noticeably increased apoptosis rates in the knee joint tissues which can be efficiently improved after M2‐sEVs treatment (Figure 2D). Taken together, M2‐sEVs can effectively improve OA progression in rats.

### 3.3 M2‐sEVs ameliorated IL‐1β‐induced inflammation and apoptosis of chondrocytes

**FIGURE 3:**
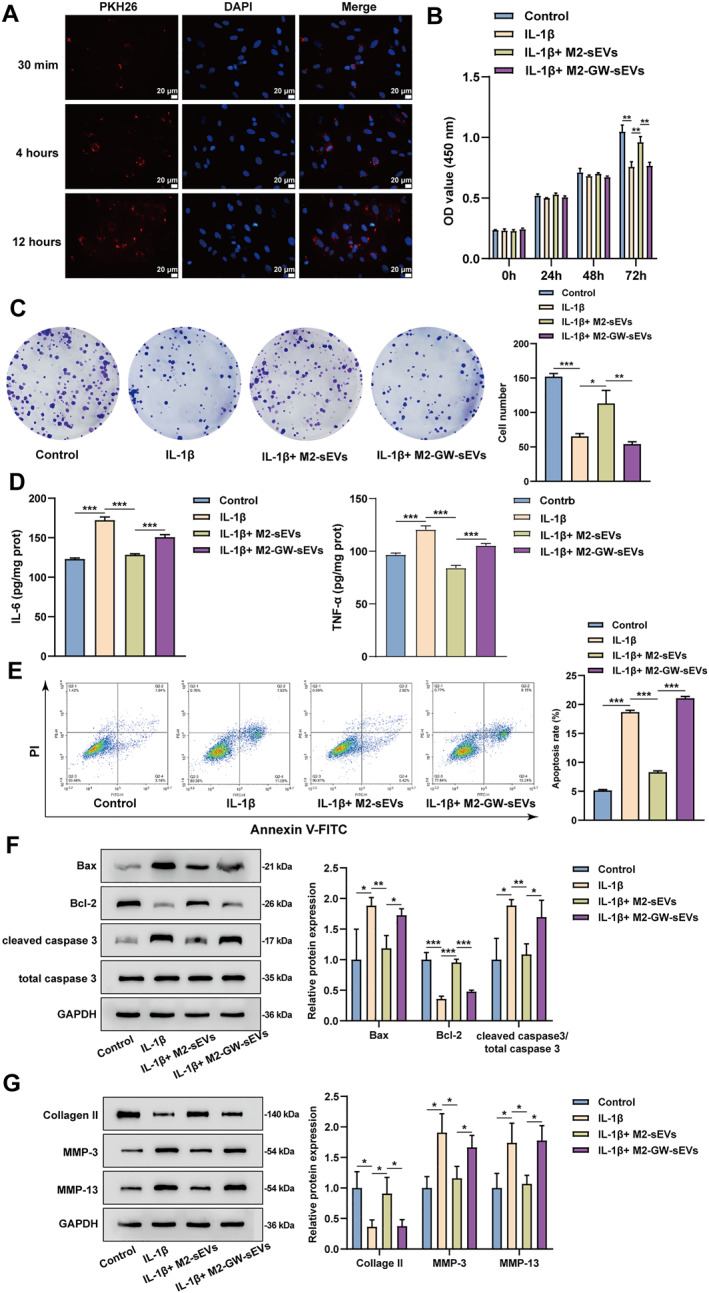
M2‐sEVs ameliorated IL‐1β‐induced inflammation and apoptosis of chondrocytes. (A) M2‐sEVs were labeled with red dye PKH26 and then co‐cultured with rat chondrocytes. Fluorescence microscopy was employed to observe the uptake of exosomes by chondrocytes; scale bar = 20 μm. (B–G) Rat chondrocytes were subjected to 24‐h stimulation with 10 ng/ml IL‐1β, followed by the addition with 50 μg/mL M2‐sEVs or equal amount of M2‐GW‐sEVs for further 24‐h culture, and then divided into four groups: control group (cells were cultured normally without any treatment), IL‐1β group (cells were induced with IL‐1β), IL‐1β+M2‐sEVs group (cells were induced with IL‐1β and M2‐sEVs), and IL‐1β+M2‐GW‐sEVs group (cells were induced with IL‐1β and M2‐GW‐sEVs). (B) The viability of chondrocytes was detected using the CCK‐8 assay. (C) The chondrocyte proliferation ability was detected using colony formation assay. (D) The level of pro‐inflammatory cytokines (IL‐6 and TNF‐α) within chondrocytes was detected using ELISA. (E) The apoptosis of chondrocytes was detected using flow cytometry. (F) The level of apoptosis‐related proteins (Bax, Bcl‐2, cleaved caspase 3, and total caspase 3). (G) ECM‐related proteins (Collagen II, MMP‐3, and MMP‐13) was detected using Western blot. *p < 0.05, **p < 0.01, and ***p < 0.001.

Subsequently, the in vitro effects of M2‐sEVs upon chondrocyte inflammation and apoptosis were explored. The M2‐sEVs uptake by chondrocytes was first observed. In short, PKH26‐labeled M2‐sEVs were subjected to co‐culture with chondrocytes. Fluorescence microscopy observed that PKH26 was distributed around DAPI fluorescence, suggesting that M2‐sEVs can be absorbed by chondrocytes (Figure 3A). Chondrocytes were induced using IL‐1β and CCK‐8 assay and colony formation assay were performed to detect cell proliferation. As indicated by the results, IL‐1β remarkably inhibited the proliferation of chondrocytes (Figure 3B–C). Moreover, the expression level of pro‐inflammatory mediators was detected using ELISA. IL‐6 and TNF‐α expressions in chondrocytes showed to be notably increased after IL‐1β stimulation (Figure 3D). Flow cytometry results of treated chondrocytes revealed that IL‐1β significantly promoted chondrocyte apoptosis (Figure 3E). The levels of apoptosis‐associated proteins (Bax, Bcl‐2, and cleaved caspase3) and ECM‐associated proteins (Collagen II, MMP‐3, and MMP‐13) within chondrocytes were detected using Western blot, suggesting that Bax, cleaved caspase3, MMP‐3, and MMP‐13 level was notably increased, whereas Bcl‐2 and Collagen II protein level was reduced within IL‐1β‐treated chondrocytes relative to normal controls (Figure 3F–G). Compared to the IL‐1β stimulation alone, treatment with M2‐sEVs dramatically increased chondrocyte activity and proliferation ability, decreased the level of proinflammatory mediators (IL‐6 and TNF‐α), and decreased the apoptosis rate, as well as remarkably elevated Bax, cleaved caspase3, MMP‐3, and MMP‐13 levels and decreased Bcl‐2 and Collagen II levels (Figure 3B–G). Additionally, to further clarify the regulatory effect of M2‐sEVs on chondrocytes, we used GW4869 to suppress the chondrocyte exosome secretion (M2‐GW‐sEVs group). To verify the inhibitory effect of GW4869 on exosomes, Western blot was applied to detect the levels of the exosomal markers and OPN in protein samples extracted from sEVs (Figure S1). After GW4869 treatment, the expression levels of OPN, CD9, CD63, CD81, and calnexin were almost undetectable in protein samples of exosomes, confirming the inhibitory effect of GW4869 on exosomes (Figure S1). M2‐GW‐sEVs treatment noticeably reversed these above changes in the IL‐1β + M2‐sEVs group, involving chondrocyte viability and apoptosis, and the expression levels of the above factors (Figure 3B–G). Taken together, M2‐sEVs can effectively improve IL‐1β‐induced inflammation and apoptosis of chondrocytes, and promote chondrocyte proliferation.

### 3.4 M2‐sEVs promoted OPN expression in OA chondrocytes

**FIGURE 4:**
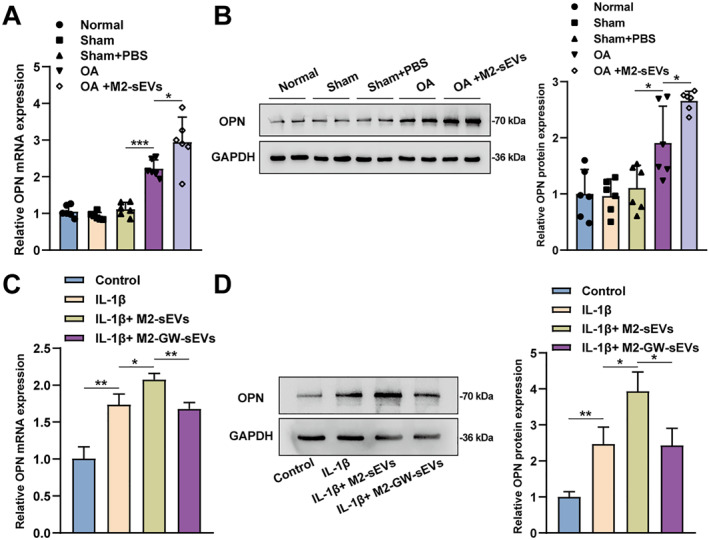
M2‐sEVs promoted OPN expression in OA chondrocytes. (A) qRT‐PCR and (B) Western blot were applied to detect OPN mRNA expression and protein level within rat knee joint cartilage tissues. (C) qRT‐PCR and (D) Western blot were used to detect OPN mRNA expression and protein level within chondrocytes. *p < 0.05 and **p < 0.01.

As previously reported, OPN is a key protective factor against cartilage injury and OA progression ,^17^, ^28^ and it is protectively elevated in OA. Therefore, whether M2‐sEVs can regulate OPN expression in OA was subsequently investigated. qRT‐PCR and Western blot were conducted to detect OPN mRNA and protein expression within rat cartilage tissue samples and chondrocytes. According to the results, rats with OA showed dramatically increased OPN expression in the knee joint tissues compared with the control rats; M2‐sEVs treatment could further increase OPN expression within rats with OA (Figure 4A–B). The in vitro experiments also showed similar results. OPN expression level within IL‐1β‐induced chondrocytes showed to be notably increased, which was further elevated after M2‐sEVs treatment, and significantly decreased after M2‐GW‐sEVs treatment (Figure 4C–D). These results suggested that M2‐sEVs can promote OPN expression in OA chondrocytes.

### 3.5 Silencing of OPN partially offset the therapeutic effects of M2‐sEVs upon OA chondrocytes

**FIGURE 5:**
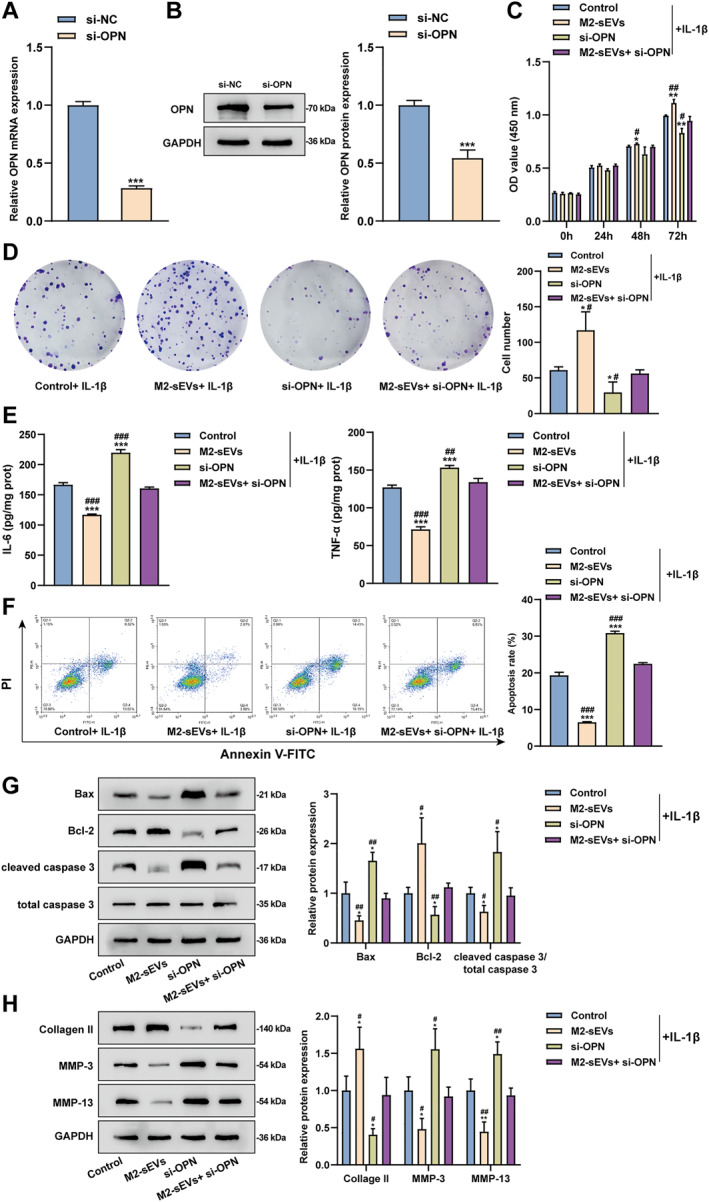
Silencing of OPN partially offseted the therapeutic effects of M2‐sEVs upon OA chondrocytes. (A) qRT‐PCR and (B) Western blot were used to detect the expression of OPN mRNA expression and protein level in chondrocytes after OPN knockdown (si‐OPN). IL‐1β‐induced chondrocytes were subjected to co‐culturing with M2‐sEVs and/or si‐OPN for 24 h. (C) The chondrocyte viability was detected using CCK‐8 assay. (D) The chondrocyte proliferation was detected using colony formation assay. (E) The expression levels of pro‐inflammatory mediators (IL‐6 and TNF‐α) within chondrocytes were detected using ELISA. (F) The chondrocyte apoptosis was detected using flow cytometry. (G) The contents of apoptosis‐associated proteins (Bax, Bcl‐2, cleaved caspase 3, and total caspase 3). (H) ECM‐associated proteins (Collagen II, MMP‐3, and MMP‐13) were detected using Western blot. A–B, ***p < 0.001, versus the si‐NC group; C‐H, *p < 0.05, **p < 0.01, and ***p < 0.001, versus control group; #*p* < 0.05, ##*p* < 0.01, and ###*p* < 0.001, versus M2‐sEVs + si‐OPN group.

Next, chondrocytes were transfected with OPN interference RNA (si‐OPN) to further validate the therapeutic effects of M2‐sEVs upon OA as confirmed by qRT‐PCR and Western blot. As indicated by the results, si‐OPN markedly inhibited OPN mRNA expression and protein level in chondrocytes relative to si‐NC (Figure 5A–B). After that, IL‐1β‐induced chondrocytes were subjected to treatment with si‐OPN and/or M2‐sEVs, suggesting that relative to the normal controls, si‐OPN treatment notably inhibited the IL‐1β‐induced chondrocyte viability and proliferation, promoted the expression levels of pro‐inflammatory mediators (IL‐6 and TNF‐α), and facilitated cell apoptosis as well as promoted the levels of Bax, cleaved caspase3, MMP‐3, and MMP‐13, and inhibited Bcl‐2 and Collagen II protein levels within IL‐1β‐induced chondrocytes. Moreover, si‐OPN can partially counteract the therapeutic effects of M2‐sEVs on IL‐1β‐induced chondrocytes (Figure 5C–H). Taken together, silencing OPN promoted IL‐1β‐induced chondrocyte inflammation and partially offset the therapeutic effects of M2‐sEVs on chondrocytes.

### 3.6 M2‐sEVs promoted OPN expression in chondrocytes and activated CD44 and the PI3K/AKT signaling pathway

**FIGURE 6:**
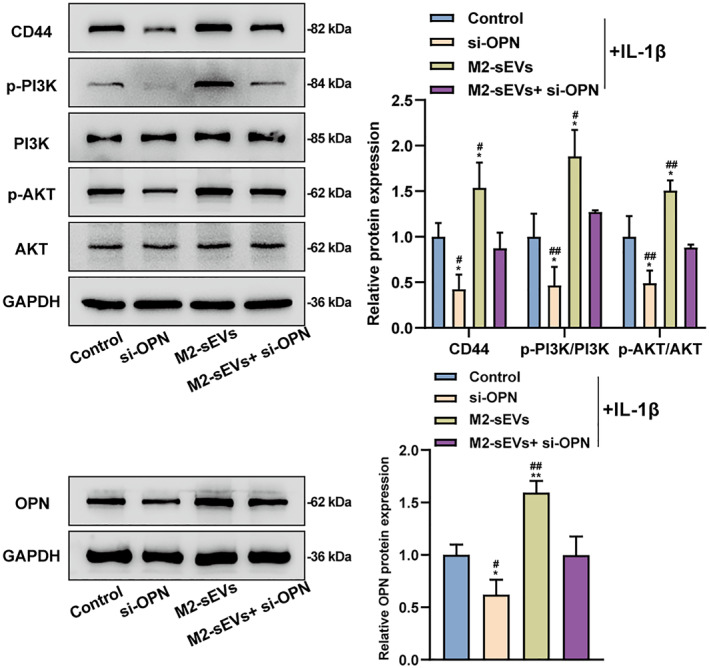
M2‐sEVs promoted OPN expression in chondrocytes and activated CD44 and the PI3K/AKT signaling. The levels of CD44 and the PI3K/AKT pathway‐related proteins (p‐PI3K, PI3K, p‐AKT, and AKT) within chondrocytes were detected using Western blot. *p < 0.05, **p < 0.01, ***p < 0.001, versus control group; #*p* < 0.05, ##*p* < 0.01, and ###*p* < 0.001, versus M2‐sEVs + si‐OPN group.

As previously reported, the OPN‐CD44 ligand‐receptor response can regulate the downstream signaling molecules mainly including the PI3K/AKT pathway.^29^ Moreover, the PI3K/AKT signaling was found to contribute to chondrocyte proliferation and apoptosis.^30^ Therefore, it was speculated that M2‐sEVs may promote OPN expression in chondrocytes to activate CD44 and the downstream PI3K/AKT signaling. The contents of CD44 and the PI3K/AKT pathway‐related proteins in chondrocytes were detected using Western blot, suggesting that relative to the normal controls, after OPN knockdown, the levels of CD44, p‐PI3K/PI3K, and p‐AKT/AKT were remarkably reduced; M2‐sEVs treatment efficiently elevated the levels of CD44, p‐PI3K/PI3K, and p‐AKT/AKT; si‐OPN can partially offset the improving effects of M2‐sEVs (Figure 6). Taken together, M2‐sEVs can promote OPN expression in chondrocytes and activate CD44 and the PI3K/AKT signaling.

## 4 DISCUSSION

OA is a degenerative and inflammatory disorder. Current treatment methods primarily involve the use of analgesics and nonsteroidal anti‐inflammatory drugs.^31^ However, these treatments focus solely on symptom relief and do not efficiently promote cartilage regeneration and repair.^32^ Recently, mesenchymal stem cell (MSC) therapy has been reported to alleviate OA symptoms and mitigate cartilage damage.^33^ It is extensively accepted that MSCs promote tissue regeneration primarily through their paracrine effects that include the secretion of growth factors, chemokines, and sEVs.^34^, ^35^ Increasing evidence has highlighted the role of macrophage‐derived sEVs in various diseases. For example, M2‐sEVs have demonstrated therapeutic effects on various inflammatory diseases,^36^, ^37^ whereas M1 macrophage‐derived sEVs have been shown to promote the progression of osteomyelitis.^38^ Therefore, macrophages‐secreted sEVs may play crucial roles in OA. The aim of this study was to explore the therapeutic effects of M2‐sEVs upon OA and the potential mechanisms.

In the present study, the therapeutic effects of M2‐sEVs upon OA were explored in both in vitro and in vivo models. OA progression is commonly associated with increased cartilage remodeling.^39^ Additionally, the expression levels of pro‐inflammatory mediators (including IL‐6 and TNF‐α) and MMPs (including MMP‐13) were promptly increased, promoting the apoptosis of chondrocytes and ECM degradation.^40^ Previous studies have demonstrated that MSC‐secreted exosomes can improve the pathological characteristics of OA by reducing inflammation, mitigating catabolic mechanistic degradation and apoptosis, and ameliorating articular cartilage destruction.^8^ Our in vivo experiments showed that M2‐sEVs treatment effectively alleviated knee cartilage damage and reduced inflammatory infiltration in OA rat models. Specifically, M2‐sEVs reduced IL‐6, TNF‐α, MMP‐3, and MMP‐13 levels while increasing Collagen II levels and reducing the number of apoptotic cells in rat knee cartilage tissues. Similar results were also observed in *vitro* experiments. M2‐sEVs were internalized by chondrocytes and remarkably attenuated IL‐1β‐induced chondrocyte apoptosis while promoting cell proliferation. Treatment with M2‐sEVs elevated Bcl‐2 levels and reduced Bax and cleaved caspase3 levels. Meanwhile, M2‐sEVs suppressed the production of IL‐6, TNF‐α, MMP‐3, and MMP‐13 while promoting Collagen II synthesis in chondrocytes. Consistent results have also been demonstrated that M2 macrophage‐derived extracellular vesicles alleviate OA by modulating macrophage polarization^11^ and promoting synovial lymphangiogenesis.^41^ The above findings demonstrated that M2‐sEVs can effectively alleviate cartilage injury, reduce inflammation and chondrocyte apoptosis, and enhance ECM synthesis in OA rat models.

After confirming the therapeutic effects of M2‐sEVs upon OA, the underlying molecular mechanism was further investigated. Our previous research has identified OPN as a key protective factor against cartilage injury and OA progression.^18^ OPN is upregulated within the cartilage tissues of patients with OA.^42^ Reportedly, during the pathological process of OA, chondrocytes will adjust the composition and metabolism of joint cartilage to maintain cartilage homeostasis under appropriate mechanical stimulation,^43^ a process that may lead to the release of OPN. Moreover, M2‐sEVs have been reported to elicit OPN expression.^19^ In this study, OPN was upregulated in the knee joint cartilage tissues of OA rat models and in IL‐1β‐induced chondrocytes. OPN expression was further upregulated following treatment with M2‐sEVs. Consistent with previous findings, OPN has been shown to mitigate OA‐related cartilage damage, promising to be a potential therapeutic agent for OA.^44^ Therefore, M2‐sEVs may exert protective effects on cartilage tissues against OA progression via promoting OPN expression. To validate the role of OPN in the therapeutic effects of M2‐sEVs, siRNA‐mediated OPN knockdown (si‐OPN) was performed in IL‐1β‐induced chondrocytes. OPN inhibition exacerbated IL‐1β‐induced chondrocyte damage as manifested by increased chondrocyte apoptosis and decreased proliferation. Additionally, OPN knockdown upregulated the levels of apoptosis‐associated proteins Bax and cleaved caspase3, decreased Bcl‐2 levels, promoted IL‐6, TNF‐ α, MMP‐3, and MMP‐13 levels, and inhibited Collagen II synthesis. Furthermore, si‐OPN partially counteracted the therapeutic effects of M2‐sEVs upon chondrocytes. Taken together, M2‐sEVs may upregulate OPN expression to inhibit chondrocyte apoptosis and inflammation while promoting chondrocyte proliferation, thereby ameliorating OA progression.

CD44 is a cell surface receptor that specifically binds to OPN, and its expression positively correlates with the severity of OA joint injury.^15^ Additionally, the OPN‐CD44 ligand–receptor interaction has been shown to regulate the activation of the downstream PI3K/AKT signaling.^44^ The PI3K/AKT signaling activation is tightly implicated in inhibiting chondrocyte apoptosis.^45^ Based on this, the roles of CD44 expression and PI3K/AKT signaling activation in M2‐sEV therapy for OA were further investigated. The results revealed that M2‐sEVs treatment notably increased CD44 expression and activated the PI3K/AKT signaling. However, silencing si‐OPN inhibited CD44 expression and suppressed the PI3K/AKT signaling; si‐OPN might partially abrogated the therapeutic effects of M2‐sEVs on OA. Taken together, M2‐sEVs promote OPN expression, which in turn enhances CD44 expression and activates the PI3K/AKT signaling, thus exerting therapeutic effects on OA.

However, the limitations of this study must be acknowledged. First, the specific mechanism by which M2 macrophages regulate the expression and secretion of OPN remain unclear. Future studies could utilize proteomics to identify proteins associated with OPN regulation in M2 macrophages. Second, as all experiments were carried out in rat models and cultured cells, the findings may not be directly translatable to humans. Further clinical trials are needed to validate the therapeutic potential of M2‐sEVs for OA. Nonetheless, the results of this study provide a solid foundation for the clinical application of extracellular vesicles in OA treatment.

**FIGURE 7:**
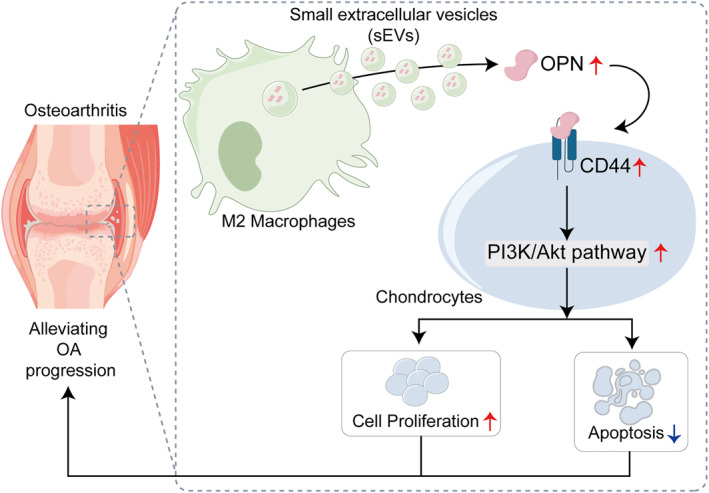
M2 macrophages secret small extracellular vesicles (sEVs) to promote OPN expression in chondrocytes, regulate CD44 and the PI3K/AKT signaling, and subsequently promote the proliferation of OA chondrocytes thereby alleviating OA development.

In summary, the present study demonstrated that M2‐sEVs effectively ameliorate cartilage tissue damage and chondrocyte injury in rats with OA. This therapeutic effect are likely mediated by upregulating OPN expression, which promotes chondrocyte proliferation and inhibits the apoptosis, inflammation, and ECM degradation. Furthermore, M2‐sEVs enhance CD44 expression and activate the PI3K/AKT signaling pathway via OPN upregulation (Figure 7). These findings suggest that M2‐sEVs hold promise as a novel therapeutic strategy for the clinical treatment of OA.

## AUTHOR CONTRIBUTIONS

**Min Tu:** Conceptualization; data curation; funding acquisition; writing—original draft. **An‐Min Liu:** Investigation; methodology. **Wei Huang:** Data curation; resources. **Dan Wang:** Software; visualization. **Hou‐Qiong Chen:** Formal analysis; validation. **Xiao‐Yuan Hu:** Project administration; supervision; writing—review and editing.

## CONFLICT OF INTEREST STATEMENT

The authors declare no conflicts of interest.

## ETHICS STATEMENT

The guidelines for the care and use of animals were approved by the Jingmen People's Hospital Medical Ethics Committee (approval No.: KY‐202304050002).

## Supporting information

Supporting information S1.

Figure S1 **Verification of the inhibitory effect of GW4869 on exosomes.** M2‐GW‐sEVs were isolated from the conditioned medium obtained after culturing M2 macrophages with 20 μg/ml GW4869 (blocking the generation of sEVs); and then western blot was employed to detect OPN, CD9, CD63, CD81, and calnexin expressions in M2‐sEVs and M2‐GW‐sEVs.

## Data Availability

The authors confirm that the data supporting the findings of this study are available within the article. All experimental data presented in this research and Supplementary Information can be obtained from figshare (https://figshare.com/s/df777f4a20af81213192).
