# Supplementary material for: Macrophages‐derived small extracellular vesicles regulate chondrocyte proliferation and affect osteoarthritis progression via upregulating Osteopontin expression
Source: J Cell Commun Signal. 2025 Apr 22;19(2):e70008. doi: 10.1002/ccs3.70008 (PMC12012988; doi:10.1002/ccs3.70008)
Supplement: Supplementary file 1 — Supporting information S1. [file CCS3-19-e70008-s001.pdf]

荆门市人民医院医学伦理委员会

Jingmen People's Hospital Medical Ethics Committee

科学研究项目审批件

Scientific Research Project Approval

伦审（科）第 202304050002 号

NO.KY-202304050002

<table>
<tr><td>项目名称
Project Name</td><td colspan="5">M2 巨噬细胞胞外囊泡传递 OPN 调控软骨细胞增殖和凋亡影响骨关节炎进展的研究
Macrophages-derived small extracellular vesicles regulate chondrocyte proliferation and affect osteoarthritis progression via upregulating OPN expression</td></tr>
<tr><td>承担专业
Major</td><td>骨科
Department of Orthopedics</td><td>承担责任
Assume responsibility</td><td>负责■参与□
Responsible■; Participant□</td><td>主要研究者
Major Investigator</td><td>涂敏
Min Tu</td></tr>
<tr><td>研究分类
Research Categories</td><td>1■ 2■ 3□</td><td>研究起止时间
Project Duration</td><td colspan="3">2023 年 6 月—2025 年 12 月
June 2023 – December 2025</td></tr>
<tr><td>研究类型
Research Type</td><td colspan="5">基础研究
Basic Research</td></tr>
<tr><td>审查意见
Review Comments</td><td colspan="5">同意■；作必要修改后同意□；不同意□；终止□；暂停已批准的试验□
Agree■; After necessary modifications, agree□; Disagree□; Terminate□; Suspend the approved experiment□</td></tr>
<tr><td colspan="6">该研究动物实验的主要内容：
本研究使用 30 只 8 周龄健康雄性 SPF 级 SD 大鼠。所有大鼠在正式实验前将进行 1 周的适应性饲养，以确保动物的健康状态。实验大鼠随机分为五组，每组 6 只，具体分组如下：①正常组（Normal）：不做任何处理，作为健康对照组。②假手术组（Sham）：进行手术，但不进行 DMM 手术。③假手术 + PBS 组（Sham + PBS）：进行假手术并注射等量 PBS 溶液。③OA 组（骨关节炎模型组）：进行 DMM 手术，诱导骨关节炎。⑤OA + M2-sEVs 组（OA + M2-sEVs）：在 OA 组基础上，从实验第 4 周起，每 5 天通过微量注射器向关节腔注射 10 μL 的 M2 巨噬细胞胞外囊泡（M2-sEVs）（10×10 颗粒/mL），共 4 次注射。所有大鼠在标准环境下饲养，环境温度为 25℃，湿度为 60%，采用 12 小时光/12 小时暗周期，提供充足的食物和饮用水。在整个实验过程中，确保大鼠的生活条件符合伦理要求，并尽量减少动物的痛苦和应激反应。所有大鼠均通过吸入麻醉（异氟烷）进行麻醉。右膝关节外科手术采用无菌操作，消毒皮肤并切开关节囊，在显微镜下进行半月板韧带切除术。假手术组仅开刀关节囊，不切除半月板韧带。手术后为防止感染，所有大鼠均给予氨苄青霉素进行预防性抗感染治疗。术后 7 天内，定期检查动物的伤口愈合情况，并确保动物在恢</td></tr>
</table>

复期内的舒适。OA + M2-sEVs 组大鼠从术后第 4 周开始，每 5 天向关节腔内注射 10 μL 的 M2-sEVs 溶液，共进行 4 次注射。注射过程中严格遵循无菌操作，减少注射引起的任何不适或感染风险。实验完成后，所有大鼠进行安乐死，收集右膝关节，进行后续实验分析。

The main contents of the animal experiments in this study are as follows:

A total of thirty 8-week-old healthy male SPF SD rats were were adaptively fed for 1 week before the formal experiment. OA was induced within the rats' right knee using destabilized medial meniscus (DMM) surgery. In short, after inhalation anesthesia with isoflurane, rat skin was prepared and the surgical area skin was disinfected with iodine. The rat was placed in a supine position, and the joint capsule medial to the patellar tendon of the right knee was incised. After surgically sectioning the medial meniscus-tibial plateau ligament with micro-surgical scissors under a microscope, the meniscus was removed. Next, the joint capsule, medial thigh muscle, and connective tissues were closed sequentially with absorbable sutures, and the surgical area skin was then closed with non-absorbable sutures. The right knee was disinfected with iodine. Rats were postoperatively administrated with ampicillin to prevent infection. The control rats were only subjected to opening the right knee joint capsule without sectioning the medial meniscus-tibial ligament. Rats were designated at random into five groups (n = 6): normal, sham, sham + PBS, OA, and OA + M2-sEVs groups. Specifically, the normal rats were fed normally and not treated in any way; the sham group rats underwent surgery but without DMM; the sham + PBS rats and OA rats were subjected to injection with an equal amount of PBS into the joint cavity; starting from the 4th week of the experiment, a micro-syringe was employed to inject intra-articularly the OA + M2-sEVs rats with 10 μL of M2-sEVs (10×10 particles/mL) every five days for four consecutive injections. Subsequently, rats were fed in a standard environment (12 h light/12 h dark cycles, 25℃, 60% humidity) and accessed to food and water ad libitum. Four weeks after the first injection of M2-sEVs, rats were euthanized, with their right knee joint harvested for the subsequent experiments.

伦理审议意见：

经我院伦理委员会审议，该研究的实验设计和方案充分考虑了安全性和公平性原则，充分体现了进行实验时的代替、减少和优化三原则，保护动物的权益，并将最大程度减轻动物的疼痛、痛苦和紧张，研究内容和研究结果不存在利益冲突。

Ethical Review Opinion:

Upon review by the Ethics Committee of our institution, the experimental design and protocol of this study have fully considered the principles of safety and fairness. The 3Rs principle (Replacement, Reduction, and Refinement) is adequately reflected in the design to minimize animal use and suffering. The rights and welfare of the animals are protected, and measures have been taken to minimize pain, distress, and anxiety. The research content and results are free from any conflicts of interest.

备注：研究分类：1=动物试验；2=动物组织或细胞试验；3=其他

Note: Research Classification: 1 = Animal Experiment; 2 = Animal Tissue or Cell Experiment; 3 = Other

荆门市人民医院医学伦理委员会

Jingmen People's Hospital Medical Ethics Committee

2023 年 04 月 17 日 (April 17, 2023)
