# Supplementary figures and images for: Macrophages‐derived small extracellular vesicles regulate chondrocyte proliferation and affect osteoarthritis progression via upregulating Osteopontin expression
Source: J Cell Commun Signal. 2025 Apr 22;19(2):e70008. doi: 10.1002/ccs3.70008 (PMC12012988; doi:10.1002/ccs3.70008)

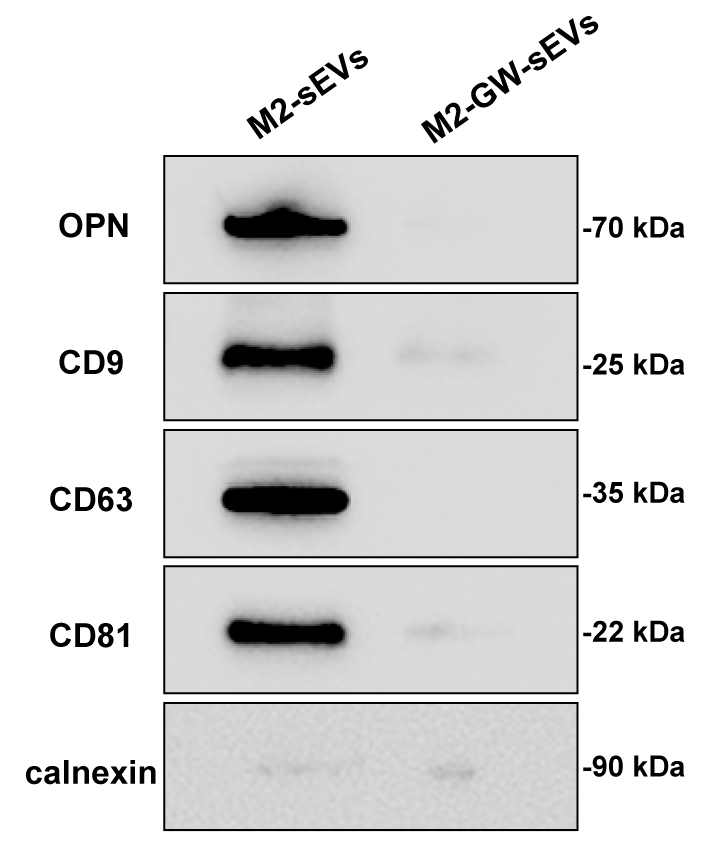

Supplement: Supplementary file 2 — Figure S1 Verification of the inhibitory effect of GW4869 on exosomes. M2‐GW‐sEVs were isolated from the conditioned medium obtained after culturing M2 macrophages with 20 μg/ml GW4869 (blocking the generation of sEVs); and then western blot was employed to detect OPN, CD9, CD63, CD81, and calnexin expressions in M2‐sEVs and M2‐GW‐sEVs. [file CCS3-19-e70008-s002.tif]
